# GAS1: A New β-Glucan Immunostimulant Candidate to Increase Rainbow Trout (*Oncorhynchus mykiss*) Resistance to Bacterial Infections With *Aeromonas salmonicida achromogenes*

**DOI:** 10.3389/fimmu.2021.693613

**Published:** 2021-07-06

**Authors:** Valérie Cornet, Trinh Dinh Khuyen, Syaghalirwa. N. M. Mandiki, Stéphane Betoulle, Peter Bossier, Felipe E. Reyes-López, Lluis Tort, Patrick Kestemont

**Affiliations:** ^1^ Research Unit in Environmental and Evolutionary Biology (URBE), Research Institute of Life, Earth & Environment, University of Namur (UNamur), Namur, Belgium; ^2^ UMR-INERIS 02 SEBIO StressEnvironnementaux et Biosurveillance des milieux aquatiques, Plateau technique mobile en cytométrie environnementale MOBICYTE, UFR Sciences Exactes et Naturelles, Université de Reims Champagne-Ardenne, Reims, France; ^3^ Laboratory of Aquaculture & Artemia Reference Center, Faculty of Bioscience Engineering, Ghent University, Ghent, Belgium; ^4^ Department of Cell Biology, Physiology and Immunology, Universitat Autònoma de Barcelona, Bellaterra, Spain; ^5^ Consorcio Tecnológico de Sanidad Acuícola, Ictio Biotechnologies S.A., Santiago, Chile; ^6^ Facultad de Medicina Veterinaria y Agronomía, Universidad de Las Américas, Providencia, Chile

**Keywords:** immune gene expression, immunostimulant, rainbow trout, bacterial challenge, beta-glucan

## Abstract

β-glucans are prebiotic and/or food additives used by the aquaculture industry to enhance the immune response of fish. Their efficiency may vary according to their origin and structure. In this study, the immunostimulant effects of two β-glucan types extracted from wild-type baker’s yeast (*Saccharomyces cerevisiae*) and its null-mutant Gas1 were investigated. Gas1 has a beta-1,3-glucanosyltransferase activity necessary for cell wall assembly. Using a positive (commercial product MacroGard^®^) and a negative control (a diet without glucans), we evaluated the immune responses and disease resistance of rainbow trout juveniles (mean weight, ~44 g) fed control, low (0.2%) and high (0.5%) doses of Macrogard^®^, Gas1, and Wild type-β-glucan after a short-term (15 days, D15) or mid-term (36 days, D36) feeding periods. We found that β-glucan supplemented diets did not affect growth performance, mortality, splenic index, or leukocyte respiratory burst activity on D15 nor D36. However, each β-glucan triggered different immune effectors, depending of the doses or length of exposure compared to others and/or the negative control. Indeed, high dose of MacroGard^®^ significantly increased lysozyme activities at D15 compared with the control and other diets (p<0.05). At D36, MacroGard β-glucan enhanced the production of lymphocytes in comparison with the control diet (p<0.05). Regarding WT β-glucan, at D36, WT-β-glucan, especially the high dose, provided the highest enzymatic activities (lysozyme and ACH50) and Ig level (p<0.01). Furthermore, on D36, Gas1 also increased lysozyme activity, Ig proportion, and some immune genes (*mcsfra*, *hepcidin*) compared with MacroGard^®^ (p<0.05). Besides, both doses of Gas1-β-glucans increased the resistance of juveniles to bacterial infection highlighted by a higher survival rate at 14 days post-challenge compared with the control and other types and doses of β-glucans (p<0.05). In conclusion, our results suggest that Gas1-β-glucan could represent a promising immunostimulant that would help to prevent diseases in aquaculture even more efficiently than other β-glucans already in use. Mode of action and particular efficiency of this new Gas1 mutant are debated.

## Introduction

Diseases are major constraints to sustainable aquaculture production, especially for intensive aquaculture systems (1–3). Indeed, fish might often be exposed to stressful conditions, eventually becoming more susceptible to microbial infections (4). For the past few decades, the traditional strategy for fish disease control in aquaculture relied on the use of antibiotics and chemical disinfectants. However, application of antibiotics can lead to the development of antibiotic-resistant bacterial strains and cause many other problems, such as environmental hazards, food safety problems, and increasing resistance of pathogens (5). The use of antibiotics can also adversely affect the health status of the fish (6, 7). Further, sub-therapeutic doses of antibiotic have been often added to aquatic feeds to promote growth, and this has further contributed to drug resistance (8). Therefore, the prophylactic use of antibiotic treatments in aquaculture has resulted in a very limited use or a ban of most compounds in top aquaculture production countries and stringent regulations on the application of antibiotics worldwide (9). This situation and recent restrictions on the use of antibiotics have promoted the use of prebiotics, probiotics (or a combination of both named synbiotics), and immunostimulant compounds (such as glucans) as preventive strategies to limit and control fish diseases (10–13).

β-glucan is a heterogeneous group of glucose polysaccharides consisting of a backbone of β(1,3)-link β-d-glucopyranosyl units with β-(1,6)-link side chains of varying length and distribution (14) and are a major structural component of fungi, bacteria, plants, algae, yeast, and mushroom cell walls. Although the cell wall β-glucans of yeast and fungi is mostly composed of 1,3-β linked glycopyranosyl residues with small numbers of 1,6 β-linked branches, oat and barley cell walls are formed of unbranched β-glucans with 1,3 and 1,4 β-linked glycopyranosyl residues, bacteria have unbranched 1,3 β-linked glycopyranosyl residues (15). Because of some similar structure with fungal or bacterial gram-negative polysaccharides, the recognition of β-glucan by host’s cell pattern recognition receptors can trigger inflammatory cascade, leading to the enhancement of the immune system (16).

Particularly, it has been reported that β-glucans bind to specific cell surface receptors of macrophages and neutrophilic granulocytes that promote the enhancement of an organism’s protective activity against infection through the activation of leukocytes, phagocytic activity, inflammatory cytokines, chemokines, reactive oxygen free radicals, increase the activity of antioxidant enzymes, and initiate the development of adaptive immunity and elimination and killing of microorganisms (11, 17–19). A wide range of studies described numerous immunostimulant effects of β-glucans in several fish species with different levels of immune stimulation, depending on fish species, β-glucans structure, dose, and duration of administration (5, 20–23).

In the rainbow trout, the potential immunomodulatory of β-glucans has been reported in several publications. Djordjevic et al. (24) showed that dietary β-1,3/1,6-glucans (level 0.2% and 0.4% diets for 37 days) decreased the expression of genes involved in acute inflammatory reactions to the inflammatory agent, whereas major parts of the immune response remained un-changed. Also, Lauridsen & Buchmann (25) reported that dietary β-1,3/1,6-glucans (0.2% diets, for 46 days) fed rainbow trout increased resistance against challenge with *Ichthyophthirius multifiliis* (white spot disease). Moreover, Skov et al. (26) reported that dietary β-1,3-glucans from *Euglena gracilis* at 1% diets, 1% biomass day^−1^ for 84 consecutive days down-regulated the expression of pro-inflammatory genes, whereas no effect of β-1,3-glucans diets on survival after *Yersinia ruckeri* challenge.

However, the effects of β-glucans on fish immune system functions can be variable, depending on the frequency, location, and length of the side-chains, which may play a role in immunomodulation. Differences in molecular weight, shape, and structure of β-glucans explain not only the differences in biological activity (15) but also fish species and administration route. Indeed, diets supplemented by a commercial β-glucans (MacroGard^®^) administrated to channel catfish (*Ictalurus punctatus*) for 4 weeks did not significantly affect several immune parameters, such as plasma lysozyme, bactericidal and hemolytic complement activities, respiratory burst of phagocytes, and the number of lymphocytes found, survival rate in fish infected with *Edwardsiella ictaluri* (27). Positive dose-related effects of β-1,3-glucans extracted from by *Saccharomyces cerevisiae* have been reported on some immune functions in rainbow trout (28). Besides, Douxfils et al. (29) found that overdoses of β-glucans (MacroGard^®^) and/or prolonged medication can lead to a non-reactive physiological status and, consequently, to a poor immune response in rainbow trout. In a previous study, Han et al. (30) demonstrated that Gas1, a β-1,3-glucans produced by the null-mutant yeasts Gas1 of *Saccharomyces cerevisiae* provided the best protection to *A. franciscana* against *Vibrio harveyi* in comparison with wild-type (WT) *Saccharomyces cerevisiae* and commercial β-1,3-glucans. The authors partly explained this observation by a different structure of the Gas1 β-1,3-glucans that has a lower degree of branching and a shorter side chain length in comparison with the two others.

In our study, we aimed to evaluate if Gas1 β-1,3-glucans could also improve fish resistance in comparison with the WT β-1,3-glucans of *Saccharomyces cerevisiae* and to a commercial one, MacroGard^®^ that effects are already well described in literature. In that purpose, we conducted a comprehensive evaluation of short- and mid-term (D15 and D36) and dose effects (low dose at 0.2% diet vs high dose at 0.5% diet) of dietary administration of different types β-glucans extracted from yeast wild or cell-wall mutants Gas1 with different particle size and chemical structure (WT- and Gas1-β-glucan) extracted by the laboratory of Aquaculture and Artemia Reference Centre (ARC, UGent) compared to a commercial β-glucan (MacroGard^®^) on the immune functions and resistance to pathogens of rainbow trout juveniles. In that purpose, blood leukocyte cells, humoral immune parameters, and immune-related gene expression in relevant immune organs, such as spleen and kidneys, will be evaluated. Moreover, the disease resistance of trout juveniles was tested by applying a bacterial challenge test based on the intraperitoneal injection of *Aeromonas salmonicida achromogenes*.

## Materials and Methods

### Experimental Fish

Feeding trial and bacterial challenge were carried out in agreement with the European and Belgian national legislation on animal welfare (Protocol number: 13197 KE). Rainbow trout juveniles (n = 315) were transported from a local fish farm (Hatrival, Belgium) to the University of Namur (in facilities of the research unit of environmental and evolutive biology) and distributed into nine fiberglass tanks of 100 L (35 fish/100 L/tank) in a recirculation system. Fish were allowed to acclimate to the new environment for 21 days.

During this period, water temperature was maintained at 13.9 ± 1.2°C by a cooling system, oxygen level averaged 11.6 ± 0.7 mg/L (aeration applied), constant photoperiod (light/dark ratio = 12:12), and fish were fed 1.5% of fish biomass, twice daily (at 9:00 am and 5:00 pm) with a specific trout diet (Coppens TROCO SUPREME-16, The Netherlands, crude protein = 48%, crude fat = 15%).

### Fish Diet and Experimental Design

After acclimation, fish (mean body weight, 44.5 ± 3.0 g) were fed by hand either a control diet (no β-glucans) or diets containing three types of β-glucans: a commercial mixture of β-glucans, MO (MacroGard^®^, Biorigin, Brazil), and two yeast β-glucan products, namely GAS1-β-glucans (GAS1) and wild type β-glucans (WT) extracted by the Laboratory of Aquaculture & Artemia Reference Center (ARC) of Ghent University, Belgium as described in Han et al. (30). For each type of β-glucans, two doses were tested (0.2% or 0.5% of the diet) for 36 days at 1% of fish biomass/day that are called further in the text: G0.2%, G0.5% for Gas1 glucan, M0.2%, M0.5% for MacroGard^®^ glucan, W0.2%, W0.5% for wild-type glucan. All diets were formulated and pelleted in the laboratory of the University of Namur (**Table 1**). Three replicate tanks were used for each experimental diet. After 15 days (short-term nutrient test, D15) and 36 days (mid-term nutrient test, D36) of feeding, six fish per tank (18 fish per experimental diet) were anesthetized in buffered (pH 7) ethyl 3-aminobenzoate methane sulfonic acid salt (98% purity, MS-222, Sigma) solution (120 mg/L). Blood was obtained by caudal vein puncture using a heparinized syringe and stored on ice in heparinized tubes. Fish were then euthanized by overdose of buffered MS-222 (240 mg/L) before decapitation. Spleen and head kidney were dissected; a part of spleen was immediately homogenized after dissection to prepare for spleen respiratory burst activity analysis. The remaining spleen and kidney samples were immediately snap-frozen in liquid nitrogen and finally stored at −80°C until analysis (RT-qPCR immune-related genes). Heparinized blood was immediately analyzed for leukocyte populations by flow cytometry, and the remaining volume of blood was then centrifuged at 7,500*g* for 10 min to collect plasma stored at −80°C until subsequent analyses (lysozyme activity, alternative complement activity, total immunoglobulin content).

**Table 1:** Ingredients and proximate composition of the experimental pelleted diet.

Ingredients	Diet* (g/kg)				
Cod fish meal^a^	350.0				
Blood meal^b^	70.0				
Wheat gluten^c^	134.0				
Cod fish oil^d^	128.0				
Starch^e^	223.6				
Carboxylmethylcellulose^e^	20.0				
α- cellulose^e^	42.4				
Mineral mix^f^	10.0				
Vitamin mix^g^	10.0				
Betaine^e^	10.0				
BHA	1.0				
BHT	1.0				
Analyzed diet composition	Crude protein (%)	Crude fat (%)	Dry matter (%)	Ash (%)	Energy (MJ/kg)
	48.00	14.75	92.16	14.30	19.8

*b-glucan doses were added in respective % of the diet reducing the levels of a-cellulose. ^a^Cod fish meal (CP-crude protein = 89%, CF-crude fat = 4.0%) provided by SNICK euroingredient NV, Ruddervoorde (Belgium). ^b^Blood meal (CP = 87.6%, CF = 0.0) as Actipro Hemoglobin, Zwevezele (Belgium). ^c^Wheat Gluten (CP = 80.0, CF = 6.0%), Roquette Freres, Lestrem (France). ^d^Sigma-Aldrich, Saint-Louis, MO, (USA). ^e^Mosselman SA, Chlin (Belgium). ^f^Mineral mix (g kg^–1^ of mix) was prepared in the lab, from (CaHPO4)2H2O, 727.77; (MgSO4)7H2O, 127.50; NaCl, 60.00; KCl, 50.00; (FeSO4)7H2O, 25.00; (ZnSO4)7H2O, 5.50; (MnSO4)4H2O, 2.54; (CuSO4)5H2O, 0.78; (CoSO4)7H2O, 0.48; (CaIO3)6H2O, 0.29; (CrCl3)6H2O, 0.13 g. ^g^Vitamin mix was provided by INVE Aquaculture Company. BHA (butylated hydroxyanisole) and BHT (butylated hydroxyl toluene) were provided by Sigma-Aldrich-Belgium.

### Bacterial Challenge

To evaluate whether β-glucan has a beneficial effect on disease resistance, rainbow trout juveniles were experimentally infected with a virulent strain of *Aeromonas salmonicida achromogenes* provided by the CER group (Centre d’Economie Rurale, Laboratoire de Pathologie des Poissons, Belgium). Bacteria were cultured in sterile Brain Heart Infusion (BHI; Sigma Aldrich, Saint-Louis, MO, USA) and incubated at 28°C for 24 h. A preliminary test infection, including various bacterial doses was performed to determine the LD50 CFU of the targeted rainbow trout population (LD50 = 3.1 × 10^7^ CFU/100 g fish body weight).

On D37, a total of 30 fish from each dietary condition (10 fish × 3 replicate tanks) were anesthetized. Then, the fish were intraperitoneally injected with a weight-adjusted dose (3.1 × 10^7^ CFU/100 g of fish body weight) of the freshly prepared *A. salmonicida achromogenes* culture and equally distributed into three 50-L tanks. The fish were confined at the animal facility (Biosafety level 2) along the infection assay. They were starved 1 day ahead of infection as well as the day of bacterial injection, and then fed the respective experimental diets until the end of the challenge test. At 35 h post-injection (D39), a total of nine fish from each dietary condition (3 fish × 3 replicate tanks) were anesthetized, and blood was sampled for subsequent immunological assays (lysozyme and alternative complement pathway activity). Levels of plasma total Ig content and spleen RBA were not determined because of limitations in this experiment. Fish were then euthanized, and spleen and kidney were sampled and immediately snap-frozen until immune gene expression analysis (RT-qPCR).

### Blood Leukocyte Populations

Blood cell populations were analyzed at D15 and D36 of dietary treatment by flow cytometry (Flow Activated Cell Sorter Calibur; Flow Cytometry System) according to Inoue et al. (31), later adapted by Mathieu et al. (32). Briefly, 10 µl of fresh heparinized blood were mixed with 1950 µl of Hanks Balanced Salt Solution (HBSS, Sigma) and 40 µl of fluorochrome DiOC6 (3,3-dihexyloxacarbocyanine, Molecular Probes, Eugene) diluted 1:10 in ethanol. The tube was mixed gently and incubated at room temperature (RT) for 10 min. The FACS was calibrated with True Count Beads diluted in HBSS, Sigma-Aldrich, Steinheim, Germany). Each blood cell population was identified by its typical location in an FL-1 v. SSC (FITC filter *versus* Side-scattered light) and FSC *vs.* SSC (Forward Scatter *versus* Side-scattered light) according to Inoue et al. (31) and Pierrard et al. (33). Four clusters were identified, thrombocyte and lymphocyte cells were gathered in the same cluster according to Pierrard et al. (33).

### Plasma Lysozyme Activity

Lysozyme activity assay was performed by the turbidimetric method of Siwicki and Studnicka (34), later adapted by Mathieu et al. (35). Briefly, 7 μl of plasma were added to 130 μl of freshly prepared *Micrococcus luteus* (Sigma-Aldrich, Saint-Louis, USA) solution (0.6 mg/ml of Na_2_HPO_4_ 0.05 M, pH 6.2) in triplicate. Absorbance corresponding to *Micrococcus luteus* lysis was measured at 450 nm for 60 min at regular intervals (5 min). One unit (U) of lysozyme was determined as an absorbance decrease of 0.001 per min.

### Plasma Hemolytic Activity of Alternative Complement Activity

Plasma hemolytic activity of alternative complement activity (ACH50) was assayed following Oriol Sunyer and Tort (36), later modified by Milla et al. (37). Briefly, 10 µl of rabbit red blood cell suspension (RRBC, Biomerieux, Mary-l’Etoile, France) suspended at 3% in veronal buffer was mixed with serial dilutions of plasma (70 µl of total volume). Hemolysis (100%) was obtained by adding 60 µl of distillate water to 10 µl of RRBC. Negative control (fresh water) was obtained by adding 60 µl of veronal buffer to 10 µl of RRBC. Samples were incubated 100 min at 27°C and centrifuged (3,000*g*, 5 min, 4°C). Then, 35 µl of supernatant was transferred to a new microplate to measure the absorbance at 405 nm. The ACH50 value was defined as the reciprocal of the plasma dilution that induced the hemolysis of 50% RRBC.

### Plasma Total Immunoglobulin Assay

Analysis of total plasma immunoglobulin content (Ig) was based on a spectrophotometric technique described by Siwicki and Anderson and later adapted by Milla et al. (37) with some modifications. Immunoglobulin was precipitated using 10,000 kDa polyethylene glycol (PEG, Sigma). Plasma was mixed with an equal volume of 12% PEG solution and shake 150 rpm for 2 h at room temperature. After centrifugation at 1,000*g* for 10 min, the supernatant was collected and assayed for its protein concentration by the method of Bradford (38). Plasma total immunoglobulin content was calculated by subtracting this value from the total protein concentration in the plasma before precipitation with PEG.

### Spleen Leukocyte Respiratory Burst Activity

Just after collection, spleen samples were conditioned in L15 medium (Sigma-Aldrich) at 4°C, and gently pressed through sterilized nylon mesh (40 µm, Dutscher) to obtain leukocyte suspensions. Then, the L15 medium-diluted samples were loaded onto Ficoll gradient (Healthcare, GE). After centrifugation (2500 rpm, 20 min, at 4°C), leukocyte suspensions were collected and washed twice in L15 medium and again centrifuged (1200 rpm, 5 min, 4°C). They were re-suspended in L15 medium and viable leukocytes were adjusted at 10^6^ cells/ml before classification of leukocyte populations (lymphocyte, macrophage, and granulocyte) by flow cytometry. RBA analysis was performed using the flow cytometry method as previously described by Chilmonczyk and Monge (39) with some modifications of Jolly et al. (40). The RBA test corresponded to an evaluation of intracellular hydrogen peroxide production following cell activation or not with phorbol 12-myristate 13-acetate (PMA). The fluorescence levels of unstimulated and PMA-stimulated cells were determined after 30 min of cell incubation (18°C in the dark) with 2′-7′dichlorofluorescin diacetate (DCFH-DA (5 µM) and DCFH-DA plus PMA (2 μg ml^−1^), respectively. The spleen respiratory burst activities were expressed in a stimulation index as the ratio between the mean fluorescence measured in stimulated cells (DCFH-DA + PMA) and the basal mean fluorescence of control (DCFH-DA only).

### RNA Precipitation and Complementary DNA Synthesis

Three pools of each experimental condition and time-point were collected to compare their gene expression profiles. Total RNA was extracted individually from the spleen and head kidney) by grounding 100 mg of the organ with a pestle until full homogenization in TriReagent (Sigma-Aldrich, St. Louis, MO). The next steps were performed according to the manufacturer’s instructions. The pellet was dried and resuspended in 200 µl of RNase-free water. Total RNA concentration was determined by Nano Drop-2000 spectrophotometer (Thermo Scientific), and the integrity was checked by Experion RNA Std Sens analysis (Bio-Rad Laboratories, Hercules, CA). Total RNA (1 µg) was used to synthesize cDNA with iScript cDNA synthesis kit (Bio-Rad Laboratories) following this protocol: priming for 5 min at 25°C, reverse transcription for 20 min at 46°C, RT inactivation for 1 min at 95°C as recommended by the manufacturer.

### Gene Expression Analysis

Real-time PCR assay was carried out to analyze the expression pattern of different genes of immunological relevance in spleen and kidney from rainbow trout stimulated with β-glucan. Samples were taken randomly from three fish of three different tanks for each treatment (n = 9) at D15 and D36 days of feeding with different doses and types of β-glucan. Samples of three randomly selected fish from each tank were pooled for each treatment (n = 3 fish per each pool). On day 37 of feeding, fish were challenged against *A. salmonicida achromogenes* and 35 h post-challenge (D39 of treatment) three fish of three different tanks for each treatment (n = 9 fish) were also collected. Samples were also taken using the same strategy from fish fed with basal diet and from non-infected fish (n = 3 fish per each pool). As a housekeeping gene, elongation factor 1 alpha (*ef-1α*) that exhibited the most stable expression (compared to *β-actin* and *18S* genes) between samples was amplified from all the evaluated samples. The gene expression of pro-inflammatory (*il-1*), macrophages (*mcsfra*), antibacterial (*Lysozyme, Hepcidin*), humoral (*mIgM*), membrane protein (*TLR_2_*), neutrophils (*mpo*), Cathelicidin (*CATH1*), T-helper (*cd4-2β*), and anti-inflammatory responses (*il-10*, *tgf-β1*) were evaluated. The list of specific primers used for gene expression analysis is given in **Table 2**, and those were designed according to Cornet et al. (41) and Khuyen et al. (42). Real-time PCR reactions were carried out with iTaq Universal SYBR Green Supermix (Bio-Rad Laboratories) using a 1:40 dilution of the cDNA for target genes or 1:1000 dilutions for *ef-1α*.

**Table 2:** Primers used for each gene expression analysis by real-time RT.

Genes	Primer sequence (5’-3’)	Accession number	Amplicon size	Efficiency
*ef-1α*	Fw: 5’-ATGCCCCCAAGTTCCTGAAG-3’	NM_001124339.1	140	1.95
	Rv: 5’-AACAGCAACAGTCTGCCTCA-3’			
*il-1*	Fw: 5’-TGAGAACAAGTGCTGGGTCC-3’	NM_001124347.2	148	2.02
	Rv: 5’-GGCTACAGGTCTGGCTTCAG-3’			
*lysozyme*	Fw: 5’-TGCCTGTCAAAATGGGAGTC-3’	NM_001124716.1	152	1.89
	Rv: 5’-CAGCGGATACCACAGACGTT-3’			
*mIgM*	Fw: 5’-AAAGCCTACAAGAGGGAGACCGAT-3’	X65263.1	128	2
	Rv: 5’-AGAGTTATGAGGAAGAGTATGATGAAGGTG-3’			
*mcsf-ra*	Fw: 5’-ATCTCCACTCATGGCGACACA-3’	AB091826	177	2
	Rv: 5’-CATCGCACTGGGTTTCTGGTA-3’			
*mpo*	Fw: 5’-GCAGAGTCACCAATGACACCA-3’	GBTD01119227	68	2
	Rv: 5’-ATCCACACGGGCATCACCTG-3’			
*il-10*	Fw: 5’-CCGCCATGAACAACAGAACA-3’	NM_001245099.1	105	1.98
	Rv: 5’-TCCTGCATTGGACGATCTCT-3’			
*tgf-β1*	Fw: 5’-GCCAAGGAGGTCCACAAGTT-3’	NM_001281366.1	146	2.06
	Rv: 5’-GTGGTTTTGATGAGCAGGCG-3’			
*cd4-2β*	Fw: 5’-AAGCCCCTCTTGCCGAGGAA-3’	AY899932	108	2
	Rv: 5’-CTCAACGCCTTTGGTACAGTGA-3’			
*vapA*	Fw: 5’-ATTAGCCCGAACGACAACAC-3’	KP184543.1	148	2.02
	Rv: 5’-CCAACACAATGAAACCGTTG-3’			

Primers were designed according to Cornet et al. (41) and Khuyen et al. (42).

Primers for target genes were used at 500 nM. The thermal conditions used were 3 min at 95°C of pre-incubation, followed by 40 cycles at 95°C for 30 s, and 60°C for 30 s. All reactions were performed using ABIprism 7300 (Applied Biosystem), and quantification was done according to the Pfaffl method (43) corrected for efficiency of each primer set. Values for each sample were expressed as normalized relative expression (NRE), calculated in relation to values of the control group, and normalized against those of the housekeeping gene *ef-1α*. The results are expressed as an average of values obtained in all pools from D15 and D36 of feeding, and D39 (after bacterial challenge test).

### Statistical Analysis

Results are presented as box plot representing the quartile distribution of the data. Statistical analyses were performed using Rstudio software (R version 4.0.3). For each timepoint, which was treated independently, the effects of the concentration, type of glucan, and their interaction were analyzed using a generalized linear model with the following code: glm (variable ~ type of glucan × concentration, family = Gaussian, data = timepoint). Post hoc comparisons (Tukey’s test) at a 5% significant level were performed on the factor (type of glucan or concentration) or their interaction (type of glucan × concentration) with general linear hypotheses and multiple comparisons for parametric models (multcomp R package) and Benjamin-Hochberg (BH) correction was applied. When two groups (from either the concentration, type of glucans or their interaction depending on the results of the glm) do not differ from each other, they will share the same letter annotation (for example “a”), and when two groups are significantly different, they will be identified by different letters (“a” and “b”). As in this study, the interaction of two factors is considered and that seven groups (control, 3 types β-glucans × 2 concentrations) are compared, it is possible that a group is annotated with more than one letter (for example “ab”).

## Results

### Specific Growth Rate, Mortality, and Splenic Index

Fish fed different β-glucan types showed no significant differences of SGR in comparison with the control diet either on day 15 (D15) or day 36 (D36) (**Table 3**). Mortality rate did not differ between controls and β-glucan treatments (**Table 3**). Furthermore, no significant changes in SI values were observed on D15 and D36 in all β-glucan diets compared with control diet (**Table 3**).

**Table 3:** Mean ( ± SD) values for specific growth rate (SGR), mortality and splenic index (SI) of trout juveniles fed with low (0.2%) and high (0.5%) doses of different beta glucan types on day 15 and day 36 of the feeding trial.

Times (Days)	Variables	Control	Macrogard		GAS1		Wild type		F_-values_	P_-values_
			M0.2%	M0.5%	G0.2%	G0.5%	W0.2%	W0.5%		
15	SGR (%/day)	1.53 ± 0.33	1.46 ± 0.26	1.72 ± 0.32	1.56 ± 0.32	1.32 ± 0.37	1.80 ± 0.01	1.60 ± 0.18	1.497	0.254
	Mortality (%)	3.8 ± 6.6	4.8 ± 8.2	2.9 ± 4.9	4.8 ± 5.9	1.0 ± 1.6	4.8 ± 5.9	1.0 ± 1.6	0.233	0.958
	Splenic index (%)	0.11± 0.02	0.11 ± 0.02	0.13 ± 0.04	0.12 ± 0.03	0.12 ± 0.02	0.11 ± 0.01	0.11 ± 0.03	0.214	0.966
36	SGR (%/day)	1.58 ± 0.06	1.54 ± 0.10	1.73 ± 0.35	1.51 ± 0.08	1.75 ± 0.34	1.36 ± 0.34	1.54 ± 0.06	0.224	0.965
	Mortality (%)	3.3 ± 5.8	0.0 ± 0.0	1.1 ± 1.9	1.3 ± 2.2	1.1 ± 1.9	3.6 ± 3.5	1.1 ± 2.0	0.586	0.736
	Splenic index (%)	0.16 ± 0.03	0.16 ± 0.04	0.18 ± 0.03	0.18 ± 0.02	0.17 ± 0.01	0.16 ± 0.04	0.17 ± 0.00	0.438	0.841

Statistical differences between dietary treatments are indicated by different lower-case letters (p < 0.05). Splenic index = weight of spleen (g)/weight of body(g). (n= 3 for SGR and mortality and n=9 for SI).

### Humoral Immune Variables

**Figure 1:**
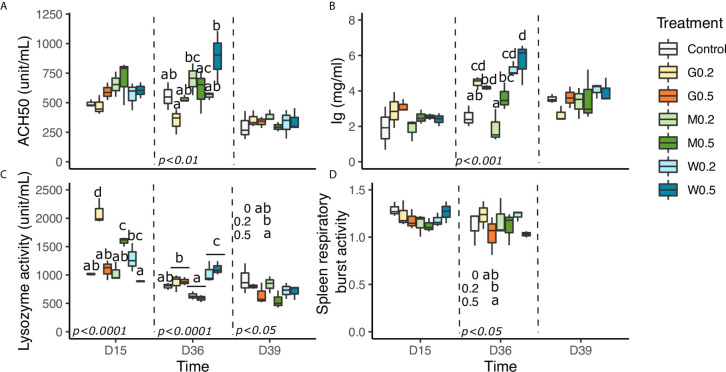
Effects of dietary beta glucan administration on humoral parameters of fish sampled during the nutrient test on D15 and D36, or 2 days after bacterial infection (D39). **(A)** Plasma hemolytic alternative complement activity. **(B)** Plasma total immunoglobulin’s content. **(C)** Plasma lysozyme activity. **(D)** Respiratory burst activity (RBA) of spleen macrophage cells. Values are expressed as mean ± SD of 18 fish/diet/day on D15, D36 and 9 fish/diet on D39. For spleen respiratory burst activity (RBA), plasma hemolytic alternative complement activity (ACH50) data are expressed as mean ± SD of 9 fish/diet/day on D15, D36. Statistical differences between groups are indicated by different letters Statistical differences, highlighted by letters, are indicated for glucan concentrations in front of 0, 0.2, and 0.5% next to the corresponding plot, for glucan type by letter above the horizontal bar grouping the two doses of the same glucan, for glucan type × concentrations interactions above each box. P values are shown at the bottom of each graph at a given timepoint (D15, D36, or D39).

On D36, the high dose of wild type β-glucan (W0.5%) significantly stimulated ACH50 level in comparison with M0.5% and G0.2%, whereas G0.2% significantly reduced it compared to the M0.2% (p<0.01, **Figure 1A**). At D15 and 2 days after bacterial injection (D39), there was no significant difference of plasma ACH50 level between treatment and control groups.

On D36, G0.2%, W0.2%, and W0.5% significantly elevated the levels of plasma total Ig (*p* < 0.001) compared to the control diet (**Figure 1B**) and M0.2%.

Concerning lysozyme parameters, on D15, M0.5% significantly improved its activity compared to the control diet and M0.2%. On the contrary, for GAS1 and wild type β-glucan, the lowest doses G0.2% and W0.2% were better to improve lysozyme activity in comparison with the control (p<0.0001) and their respective high dose (**Figure 1C**). At D36, we only observed a type of glucan effect, where WT exhibited a significant highest lysozyme activity, followed by Gas1 and then MacroGard (p<0.0001). After the bacterial infection, an effect of the concentration of β-glucan was highlighted, with a higher activity with 0.2% in comparison with 0.5% of β-glucan (p<0.05).

Eventually, levels of respiratory burst activity (RBA) of spleen lymphocytes (as for macrophage cells) did not significantly differ whatever the β-glucan administration doses and types on D15, and an effect of the concentration of β-glucan was highlighted, with a higher RBA in 0.2% compared to 0.5% (p<0.05) (**Figure 1D**).

### Blood Leukocyte Cell Proportions

**Figure 2:**
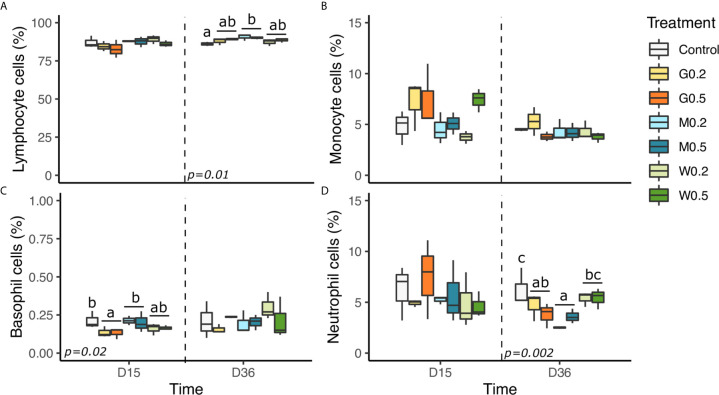
Effects of dietary beta glucan administration on blood leukocyte cell proportions of fish sampled during the nutrient test on D15 and D36. **(A)** Lymphocyte + thrombocyte cells proportions. **(B)** Monocyte cells proportion. **(C)** Neutrophil cells proportion and **(D)** Basophil cells proportion in total blood leukocyte cells population. Values are expressed as mean ± SD of 18 fish/diet/day on D15, D36. Statistical differences, highlighted by letters, are indicated for glucan concentrations in front of 0, 0.2, and 0.5% next to the corresponding plot, for glucan type by letter above the horizontal bar grouping the two doses of the same glucan, for glucan type × concentrations interactions above each box. P values are shown at the bottom of each graph at a given time point (D15, D36, or D39).

Blood leukocytes were composed of high percentages of lymphocytes followed by those of neutrophils, monocytes, and basophils (**Figures 2A–D**). Lymphocyte proportion was not modified at D15; however, on D36, lymphocyte proportions were higher with MacroGard β-glucan in comparison with the control (p<0.01).

On D15, the type of β-glucan significantly influenced the proportion of basophils with the highest quantity in fish fed MacroGard and control diets in comparison with Gas1 (p<0.05).

Finally, the type of β-glucan also significantly affected neutrophils proportion of fish as MacroGard diet reduced it in comparison of WT and the control diet.

### Immune Gene Expression

#### Pro- and Anti-Inflammatory Gene Expressions

**Figure 3:**
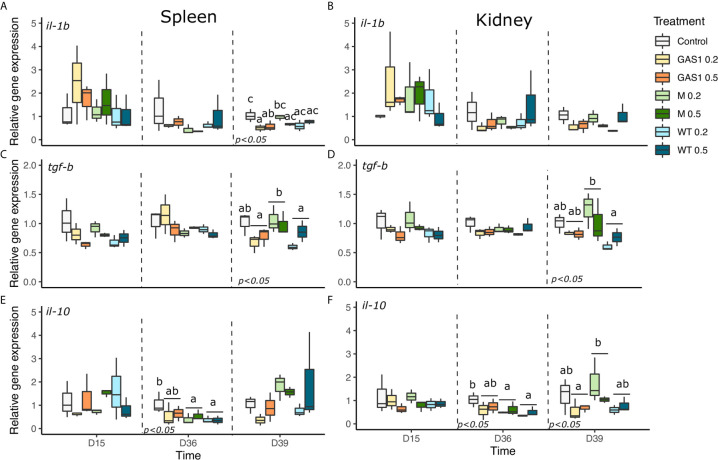
Effects of dietary β-glucan on pro-inflammatory gene expressions in spleen and kidney of fish sampled during the nutrient test on D15 and D36, or 2 days after the bacterial challenge (D39). **(A–F)** are expression levels of *il-1β*, *tgf-β1*, and *il-10* in spleen and kidney, respectively. Relative transcript (mRNA) levels were determined by real-time RT-PCR and normalized by the arithmetic mean of *ef-1α* expression. Values are expressed as mean ± SD of three pool samples per experimental condition (3 fish per pool sample). Statistical differences, highlighted by letters, are indicated for glucan concentrations in front of 0%, 0.2%, and 0.5% next to the corresponding plot, for glucan type by letter above the horizontal bar grouping the two doses of the same glucan, for glucan type × concentrations interactions above each box. P values are shown at the bottom of each graph at a given time point (D15, D36, or D39).

Expression levels of both pro-inflammatory (*il-1β*) and anti-inflammatory (*tgf-β1*, *il-10*) genes were evaluated in spleen and kidney organs of fish sampled after short-term (D15) or mid-term β-glucan administration, also the expression of some immune genes was recorded on D39 after bacterial challenge (**Figures 3A–F**).

In the spleen, no significant effect was observed at D15, whereas only the type of β-glucan affected the expression of *il-10*, with a significant decrease in MacroGard and WT diets in comparison of the control diet (*p* < 0.05, **Figure 3E**).

On D39, after bacterial challenged, G0.2% and G0.5% diets significantly reduced *il-1β* gene expression compared to the control (p<0.05, **Figure 3A**). Meanwhile, MacroGard diet up-regulated *tgf-β1* gene expression in comparison with the Gas1 and WT diet (p<0.05, **Figure 3C**).

In kidneys, MacroGard and WT diets significantly reduced *il-10* gene expression in comparison of the control diet on D36 (p<0.05; **Figure 3F**). After bacterial challenge, only the type of β-glucan affected *tgf-β1* and *il-10* gene expression compared to the control (p<0.05, **Figures 3D, F** **)**. Indeed, MacroGard diet significantly enhanced the expression of *tgf-β1* and *il-10* compared, respectively, to WT and Gas1 diets.

#### Expression of T-Helper, mIgM, Complements C_3,_ mcsfra, and TLR_2_ Genes

**Figure 4:**
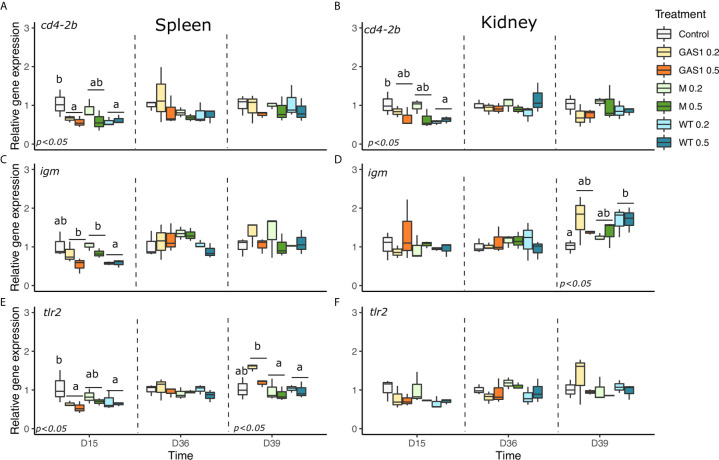
Effects of dietary beta glucan on T-helper (*cd4-2β*), humoral (*mIgM*) and membrane protein (*TLR_2_*) genes expressions in the spleen and kidney of fish sampled during the nutrient test on D15 and D36, or 2 days after the bacterial challenge (D39). **(A–F)** are expression levels of *cd4-2β*, *mIgM*, *TLR_2_*_in_ spleen and kidney, respectively. Relative transcript (mRNA) levels were determined by real-time RT-PCR and normalized by the arithmetic mean of *ef-1α* expression. Values are expressed as mean ± SD of 3 pool samples per experimental condition (3 fish per pool sample). Statistical differences, highlighted by letters, are indicated for glucan concentrations in front of 0, 0.2, and 0.5% next to the corresponding plot, for glucan type by letter above the horizontal bar grouping the two doses of the same glucan, for glucan type × concentrations interactions above each box. P values are shown at the bottom of each graph at a given time point (D15, D36, or D39).

The expression levels of T-helper genes (*cd4*-2β), membrane immunoglobulin M (mIgM), *complement C3*, *macrophage colony-stimulating factor receptor a (mcsfra)*, and *Toll like receptor* (*tlr2*) genes were determined in spleen and kidney samples (**Figure 4**).

**Figure 5:**
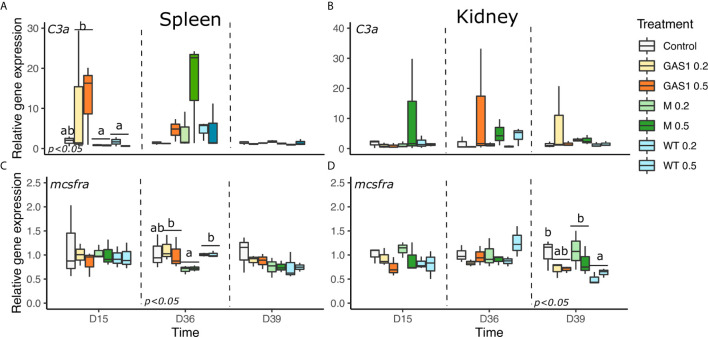
Effects of dietary beta glucan on C3a complement (*c3a*) and macrophage colony-stimulating factor receptor a (*mcsfra*) genes expressions in the spleen and kidney of fish sampled during the nutrient test on D15 and D36, or 2 days after the bacterial challenge (D39). **(A–D)** are expression levels of *c3a* and *mcsfra*_in_ spleen and kidney, respectively. Relative transcript (mRNA) levels were determined by real-time RT-PCR and normalized by the arithmetic mean of *ef-1α* expression. Values are expressed as mean ± SD of three pool samples per experimental condition (3 fish per pool sample). Statistical differences, highlighted by letters, are indicated for glucan concentrations in front of 0, 0.2, and 0.5% next to the corresponding plot, for glucan type by letter above the horizontal bar grouping the two doses of the same glucan, for glucan type × concentrations interactions above each box. P values are shown at the bottom of each graph at a given time point (D15, D36, or D39).

In spleen, after 15 days, *cd4-2β* and *tlr2* gene expressions were modified by the type of β-glucan with a repression observed in fish-fed Gas1 and WT diets compared with the control group (p<0.05, **Figures 4A, E** **)**. Similarly, Gas1 and Macrogard diets enhanced the expression of *mIgM* in comparison to WT diet at D15. In addition, Gas 1 diet also significantly raised the expression of *C3a* in comparison with MG and WT diets (**Figure 5A**). On D36, only *mcsfra* was modulated by the type of β-glucan with an increase of its expression with Gas1 and WT diets (*p* < 0.05, **Figure 5C**). After bacterial injection (D39), only *tlr2* was affected by the type of β-glucan as fish-fed Gas1 diet had a significant higher expression level compared with MacroGard and WT diets (p<0.05, **Figure 4E**).

In kidneys, WT diet significantly repressed *cd4-2β* gene expression compared with the control on D15 (*p* < 0.05; **Figure 4B**) on D15. On D36, no gene expression was modulated by the diets, while after bacterial infection, only *igm* and *mcsfra* expression was changed by the type of β-glucan in the diet. Indeed, WT diet significantly increase *igm* expression compared with the control (*p* < 0.05, **Figure 4D**), whereas it repressed *mcsfra* expression in comparison of control and MG diets (*p* < 0.05, **Figure 5D**). There was no change of *tlr2* and *C3a* gene expression in kidneys on D15, D36, and D39 after β-glucan treatments (**Figure 5B**).

#### Antibacterial Gene Expressions

**Figure 6:**
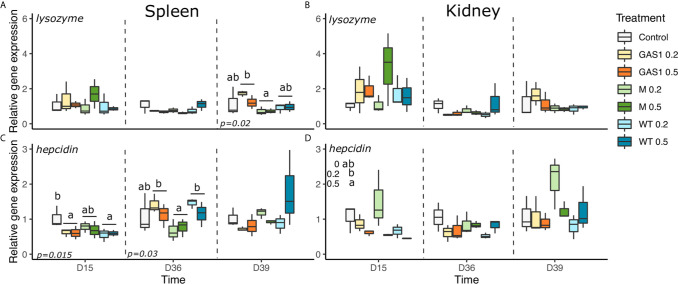
Effects of dietary beta glucans on *C-type lysozyme* and *Hepcidin* genes expression in the spleen or kidney of fish sampled during the nutrient test on D15 and D36, or 2 days after the bacterial challenge (D39). **(A)** *lysozyme* gene expression in spleen. **(B)** *lysozyme* gene expression in kidney. **(C)** *Hepcidin* gene expression in spleen. **(D)** *Hepcidin* gene expression in kidney. Relative transcript (mRNA) levels were determined by real-time RT-PCR and normalized by the arithmetic mean of *ef-1α* gene expressions. Values are expressed as mean ± SD of three pool samples per experimental condition (3 fish per pool sample). Statistical differences, highlighted by letters, are indicated for glucan concentrations in front of 0%, 0.2%, and 0.5% next to the corresponding plot, for glucan type by letter above the horizontal bar grouping the two doses of the same glucan, for glucan type × concentrations interactions above each box. P values are shown at the bottom of each graph at a given time point (D15, D36, or D39).

**Figure 7:**
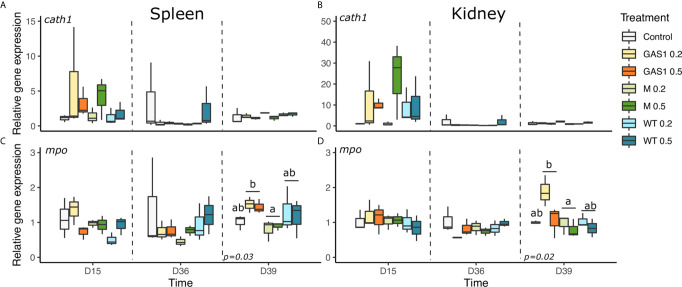
Effects of dietary beta glucans on *Cathelicidin* and *Myeloperoxidase* (*MPO*) genes expression in spleen or kidney of fish sampled during the nutrient test on D15 and D36, or 2 days after the bacterial challenge (D39). **(A)** *CATH1* gene expression in spleen. **(B)** *CATH1* gene expression in kidney. **(C)** *MPO* gene expression in spleen. **(D)** *MPO* gene expression in kidney. Relative transcript (mRNA) levels were determined by real-time RT-PCR and normalized by the arithmetic mean of *ef-1α* gene expressions. Values are expressed as mean ± SD of 3 pool samples per experimental condition (3 fish per pool sample). Statistical differences, highlighted by letters, are indicated for glucan concentrations in front of 0%, 0.2%, and 0.5% next to the corresponding plot, for glucan type by letter above the horizontal bar grouping the two doses of the same glucan, for glucan type × concentrations interactions above each box. P values are shown at the bottom of each graph at a given time point (D15, D36, or D39).

Expression of antibacterial genes (*C-type lysozyme, Hepcidin, Cathelicidin*, and *Myeloperoxidase*) were evaluated in the kidneys and spleen on D15, D36, and D39 (**Figures 6A–D** and **7A–D**).

In spleen at D15, Gas1 and WT diets caused a significant decrease of *Hepcidin* gene expression compared with the control diet (p=0.015, **Figure 6C**). On the contrary, at D36, *hepcidin* expression was higher in Gas1 and WT diet in comparison with the respective levels of fish fed MG diet (p<0.05, **Figure 6C**). After the bacterial infection, only *lysozyme* and *mpo* genes were affected by the type of β-glucan with an increase of their expression with Gas1 in comparison with MG diet (*p* < 0.05; **Figures 6A** and **7C**).

In kidneys, there was no change of *lysozyme* and *cathelicidin* gene expression on D15, D36, and D39 after β-glucan treatments (**Figures 6B** and **7B**). Regarding *hepcidin* gene expression, it was only modulated on D15 by the concentration of the compound with a higher expression of the gene with the lowest dose (*p* < 0.05; **Figure 6D**).

### Survival Rate

**Figure 8:**
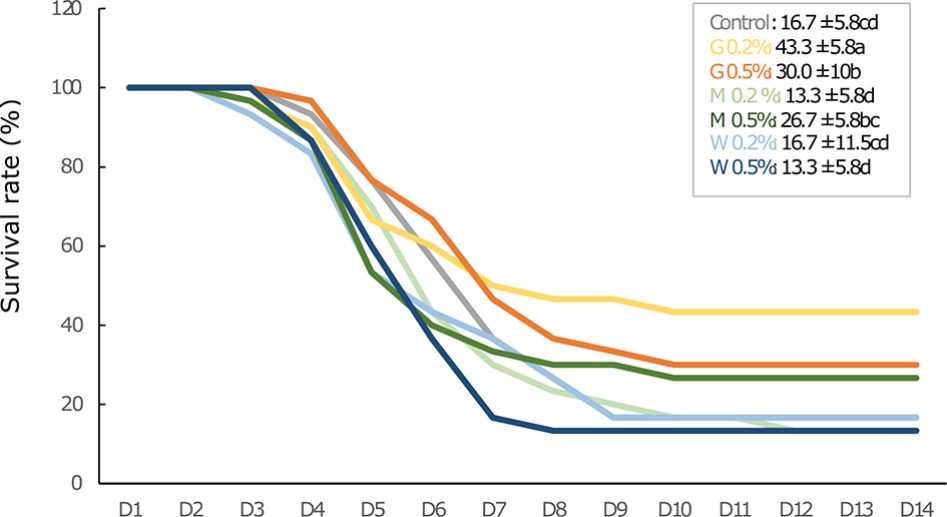
Survival rate profile in rainbow trout juveniles fed with different types of beta glucan at low (0.2% diets) or high doses (0.5% diets) and challenged with Aeromonas salmonicida achromogenes (3.1 × 107 CFU/100g fish body weight) for 14 days. Statistical differences, highlighted by letters, are indicated for glucan concentrations in front of 0, 0.2, and 0.5% next to the corresponding plot, for glucan type by letter above the horizontal bar grouping the two doses of the same glucan, for glucan type x concentrations interactions above each box. P values are shown at the bottom of each graph at a given time point (D15, D36, or D39).

The mortality rate increased earlier and more rapidly in trout fed with the control and most of β-glucan diets during the first week of infection, except for fish fed with low dose of GAS1 β-glucan (G0.2%) (**Figure 8**). The mortality of fish fed with G0.2% was reduced from day 5 and stopped on the 10th day after bacterial injections, whereas other β-glucan diets were stopped later between the 10th and 12th day post-infection. The survival rates were significantly higher in fish fed G0.2% (43.3%) and G0.5% (30.0%) than in controls (16.7%) 14 days after bacterial injection (F = 6.5, p = 0.0019; **Figure 8**), followed by M0.5% (26.7%). The higher mortality rates were observed in M0.2% and W0.5%.

## Discussion

In this study, rainbow trout juveniles were fed with a control diet or enriched diets with Macrogard-β-glucan, GAS1- β-glucan, and WT-β-glucan at low (0.2% diet) and high doses (0.5% diet), and fish were sampled after short-term (15 days) and mid-term (36 days) feedings. After 15 and 36 days of treatment, β-glucan diets did not significantly modify specific growth rate (SGR), mortality, or splenic index of treated fish in comparison with the control group. This result may be due to the duration of the experiment that was not long enough to induce a significant modification of growth performances. However, this was not the targeted effect in this study as apart from the effects on growth performance, β-glucans are mainly used for their immunostimulant capacities and previous studies highlighted the importance of the quality and structures of glucans in the activation of immune cells (22, 23, 44, 45).

In a previous paper, Han et al. (30) already demonstrated that Gas1 glucan, which was produced in their laboratory, could induce a higher survival of *Artemia franciscana* against *Vibrio harveyi* infection. The authors hypothesize that the structural features of β-glucans could be responsible for this protection, as Gas1 had lower degree of branching and shorter side chain length than WT glucan. In the present study, the capacity to trigger different immune responses of the two extracted β-glucans in the Ghent’s laboratory (GAS1 and WT) and the commercial control (MacroGard^®^) was demonstrated.

### MacroGard: The Well-Known Commercial β-Glucans

MacroGard is a well-known commercial β-glucan that was already proven to exert several immune stimulation in fish (29, 46, 47). In the present study, we showed that the high dose (0.5%) of Macrogard β-glucan (M0.5%) was better than the lower dose (M0.2%) to protect fish against bacterial infection but also to induce some effectors of the innate immune system. Indeed, on D15, M0.5% significantly stimulated plasma lysozyme activity. Lysozyme is an enzyme that possesses a lytic activity against both Gram-positive bacteria and Gram-negative bacteria and is known to activate the complement system and phagocytes and its activity reflects the activation of innate immunity of fish (48). Several studies have shown that Macrogard β-glucan could stimulate lysozyme activity. Particularly, dietary with this β-glucan at 0.2% and 0.3% increased lysozyme activity on Persian sturgeon juvenile (47) or sea bass-fed Macrogard (0.1%) diet for 30 days had a significant increase of serum lysozyme activity (46).

However, most of significant immune modulations induced by this β-glucan were observed on D36 and D39 (after bacterial infection) and were independent of the doses, except for M0.2% that significantly increased plasma alternative complement activity (ACH50) on D36 compared with G0.2%. This alternative complement activity plays a major role in the innate immune response by destroying the cell surface membranes of pathogens by creating pores and opsonizing the pathogens for destruction by increased phagocyte uptake, through ligand-receptor interactions between the two cell surfaces (49). In addition, on D36, MacroGard β-glucan enhanced the production of lymphocytes in comparison with the control diet. Siwicki et al. (50) already demonstrated the proliferative response of pronephros lymphocytes that was shown to be stimulated by mitogens.

Adversely, on D36, some immune parameters, such as lysozyme activity, immunoglobulin production, neutrophils proliferation, and genes involved in macrophage production (*mcsfra*) or in antibacterial activity (*hepcidin*) were down-regulated by the supplementation with MacroGard in comparison with Gas1/WT β-glucans or the control. Falco et al. (51) reported that treatment with dietary MacroGard β-glucan administered daily to carp for 14 days prior to infection in generally down-regulated the expression of several measured genes when compared to their corresponding control. In our study, the decreased were observed mostly in comparison with Gas1 and WT β-glucans potentially suggesting that MacroGard could induced a weakening of the immune system after a shorter period of supplementation. Nevertheless, a bacterial challenge was essential to validate this hypothesis.

After bacterial infection on D39, M0.5% diet slightly but not significantly improved fish survival after the infection to the pathogen *A. salmonicida achromogenes* in comparison to the control (26% vs 16%) while the survival in fish fed M0.2% was worse than the control diet. Although some immune genes involved in anti-inflammatory responses (i.e., *Tgfb* and *il-10*) were enhanced in comparison with Gas1 and/or WT β-glucans, several other essential in antibacterial activity (*lysozyme, mpo*) and Toll-NFkb pathway (*tlr2*) were repressed. Altogether, those results could support the hypothesis of a weakening of the innate immune system when the organism is exposed to such a high dose of β-glucan for a long period. Indeed, it has been reported that overdoses of β-glucan and/or prolonged medication can lead to non-reaction physiological status of rainbow trout (29). The decrease of several immune genes expression in this study may be explained by the high doses of β-glucan used in this experiment leading to high levels of butyric acid production in the guts by the fermentation of *bifidobacterium* and lactic acid producing bacteria. On the other hand, butyric acid is also a histone deacetylase (HDAC) inhibitor, such as HDAC1, HDAC2, HDAC3, and HDAC8, which inhibits the function of histone deacetylase enzymes leading to deacetylase (52). The decrease of HDCA causes the loss of structures of chromatin due to the decrease of electrostatic attraction between histone and DNA (52).

Therefore, our results suggest that MacroGard β-glucan supplementation in trout should be at least 0.5% of the diet and should be restricted to 15 days to avoid immune weakening and improve fish survival in case of bacterial infection.

### GAS1: The Best β-Glucan to Improve Resistance to *A. salmonicida achromogenes*

As described by Han et al. (30), Gas1 is a β-glucan produced by the null-mutant yeasts Gas1 of *Saccharomyces cerevisiae* (isogenic deletion strains derived from baker’s yeast strain BY 4741). This β-glucan possesses a lower degree of branching and shorter side chain length and exerted the most prominent *V. harveyi*-protective effects in *A. franciscana* (30). Similarly, in our study, low and high doses of Gas1-β-glucan (G0.2% and G0.5% diet) significantly increase trout immune system with different cellular or molecular immune effectors stimulated at the three timepoints (*i.e*., D15, D36, and D39).

On D15, Gas1 β-glucan significantly decreased basophils proportions after a short-term exposure compared to control and MacroGard diets. In previous studies, the modulation capacity of the leukocyte production by β-glucans was already observed (53–55). In mammals, basophils are immune cells known to be involved in allergic process and antiparasitic immunity, and release inflammatory mediators, such as histamine, by degranulation and induce inflammation (56). In Teleost, although their function remains poorly described, basophils were proved to be activated in an antibody-dependent manner (bounding with IgM induced degranulation) and the released granules exhibited the capacity to induce the migration of various leukocytes (57). Furthermore, the expression of *cd4* coding for a co-receptor of the T cell receptor (TCR) that assists in the communication with antigen-presenting cells and *tlr2* were also repressed in fish fed Gas1. This repression of basophils proliferation and *cd4* with Gas1 could alter inflammatory processes, but further investigation would be necessary to evaluate to which extent. Nevertheless, Gas1 diet stimulated the activity of lysozyme and the expression of C3a involved in the alternative complement pathways in comparison to MacroGard and WT β-glucan potentially indicating a better response of innate immune system on D15.

On D36, Gas1 also increased lysozyme activity, Ig proportion and some immune genes involved in macrophage production (*mcsfra*) and antibacterial activity (*hepcidin*) in comparison with MacroGard β-glucans. As already described for MacroGard β-glucan, those results suggest the induction of some effectors of the immune system but triggered by different molecular pathways. Surprisingly, in another study, brood rainbow trout fed 0.2% MacroGard β-glucan diet for two months also exhibited a significant increase of lysozyme activity and total Ig (54). Here, only Gas1 modified those immune parameters but this could be explained by the duration of feeding or the age of the fish at the moment of the experiment. Besides, the increase of plasma total Ig was also observed with both low and high doses of Gas1 β-glucan on D36. Immunoglobulins are complex glycoproteins that play critical functions in innate and adaptive immunity thanks to their capacity of recognition, binding, and fixation of cells (58).

In the context of a bacterial intrusion, those Ig could help to cope with the infection, and could be one of the immune effectors responsible for the results obtained on rainbow trout juvenile resistance to *A. salmonicida* pathogens at D39. Indeed, as already observed in Han et al. (30), low and high doses of Gas1-β-glucan (G0.2% and G0.5% diet) exhibited the best protective effect against the pathogen. Regarding the cumulative survival rate 10 days after bacterial challenge, fish fed with G0.2% displayed the highest survival rate with 43.3%, followed by G0.5% with 30% and fish fed control diet at 16.7%. Meanwhile, there was no difference in survival rate for fish fed low and high doses of Macrogard β-glucan, Wild type (WT)-β-glucan diets in comparison to those of the control. Numerous studies have reported the immunomodulation potential of β-glucan in fish disease resistance and the available results vary greatly depending on the fish species, doses, and administration modes (14, 28, 59–61). In the present study, in addition to higher amount of Ig, the immune protection of fish fed G0.2% and G0.5% may be related to a differential expression of several innate immune genes (*lysozyme, tlr2*). Particularly, on D39, myeloperoxidase gene was still enhanced after 35 h post-infection. This enzyme present in neutrophils was shown to exert a bactericidal activity by producing hypochlorous acid (HOCl) that oxidizes key components of the invading pathogens (62). Therefore, the higher *mpo* gene expression could have induced a higher production of the enzyme leading to a higher oxidant capacity to deal with the bacterial infection in fish fed Gas1 diet. Surprisingly, we also observed that several genes related to inflammatory process (*il1b*, *il10*, *tgfb*) were repressed after 35 h of infection with the low dose of Gas1, and could be explained by a modified dynamic of gene expression over time. Indeed, in a previous study, we have shown that infection of zebrafish by *A. salmonicida achromongenes* stimulated the expression of il6 at 6 h post-infection (hpi) but was already repressed after 24 hpi, and this decrease depended on the previous infections applied on the zebrafish (63). Altogether, our results suggest that Gas1 is an efficient β-glucan that should be used at a dose of 0.2% of the diet and can be given for a month although it could have provided an even better protection against pathogen after 15 days of feeding. The high dose at 0.5% can also be used but it exhibited some immune weakening as the survival was reduced compared to the low dose at 0.2%.

### WT-β-Glucans: Similar Immune Stimulation Than Gas1, but Worst Resistance to Pathogens

This last β-glucans produced from wild-type yeast *S. cerevisiae* differs from Gas1 by its longer size and higher degree of branching. Our results of measured parameters highlighted substantial differences in the modulation of the immune systems at the three timepoints. First, on D15, W0.5% exhibited the lowest lysozyme activity compared with other diets. In addition, this β-glucan significantly reduced the expression of *cd4* and *hepcidin* in comparison with the control diet. On D36, W0.5% increased ACH50 and lysozyme activities, Ig production, and repressed one immune gene (*il-10*). Surprisingly, although the stimulation of these immune features appeared even more enhanced in WT-β-glucan than in Gas1, W0.2% and W0.5% provided no protection against *A. salmonicida achromogenes* in comparison with the control or MacroGard β-glucan at both doses. Other studies already reported that dietary β-glucans (0.1%, 0.2%, and 0.3% in diets) had no significant effect on survival of juvenile Persian sturgeon (47) or that the administration of dietary β-1,3-glucan from *Eulena gracilis* (1% in diet) had no effect on the survival rate of unvaccinated or vaccinated rainbow trout with *Y. ruckeri* after 70 or 84 days of treatments (26). In the present experiment, the results might indicate that the stimulation of ACH50 and lysozyme activities, Ig and monocyte production was not in itself sufficient in this case to protect the fish against bacterial infection. This lack of protection could be due, as already suggested for Macrogard β-glucan, to an overstimulation of the immune system, that would be too weak after 36 days of feeding trial to respond properly to an infection. Nevertheless, it could also be due to *mpo* in neutrophils or other bactericidal enzymes that activities would be more efficient in fish fed Gas1 diet than fish fed other β-glucan diets. Further studies should be performed to exclude the influence of the doses and duration on the absence of protection by testing lower and higher doses (for example 0.1 and 1%) with a bacterial challenge at 15 days of feeding. However, as previously suggested by Han et al. (30), one explanation to this huge difference in fish protection could be because of the size and degree of branching. Bohn et al. (45) indicate that the activation of the immune cells needs the presence of some structural features, such as β-1,3-linkages in the main chain and β-1,6 or β-1,4 branch of the glucan. Finally, it is possible that their structure influences their absorption in the intestine and the binding to the receptor, and this should be further investigated to understand why Gas1 is such more efficient to protect fish against pathogen infection.

## Conclusions

In this study, the diet supplemented with 0.2% of GAS1-β-glucans was the most effective to protect rainbow trout juveniles against a bacterial infection with *A. salmonicida achromogenes.* After short-term β-glucan feeding (D15), the immune system of rainbow trout juveniles differentially responded to the two β-glucan doses tested depending on the type of β-glucans. Regarding lysozyme enzyme, low doses of Gas1 and WT (G0.2% and W0.2%) were better to stimulates its activity, while M0.5% exhibited the highest activity in comparison with M0.2%. On D36, independently of the doses, MacroGard supplementation resulted in the down-regulation of several immune parameters such as lysozyme activity, immunoglobulin production, neutrophils proliferation, and immune genes (*mcsfra*, *hepcidin*) in comparison with Gas1 diet potentially suggesting a weakening of the immune system after a shorter period of supplementation confirmed by the low survival rates of fish fed with this diet. Besides, considering the difference of survival rate between Gas1 and WT fed fish, our results suggest that the structure of the β-glucan with a lower degree of branching and shorter side chain length could potentially play a substantial role in the efficiency of the immuno-stimulation. To conclude, in the context of a mid-term feeding (36 days), it appeared that Gas1 β-glucan represents the best immunostimulant as MacroGard already exhibited some immune weakening.

## Data Availability Statement

The raw data supporting the conclusions of this article will be made available by the authors, without undue reservation.

## Ethics Statement

The animal study was reviewed and approved by Ethical commitee of Namur University, Protocol number: 13197 KE.

## Author Contributions

Ideas: SM, PB, LT, and PK. In vivo experiment: VC, TK, SM, SB, and FR-L. Data generation: TK, VC, FR-L, and SB. Data analysis: VC and TK. Manuscript preparation: VC, TK, SM, SB, PB, FR-L, LT, and PK. All authors contributed to the article and approved the submitted version.

## Funding

The authors thank to Belgian Science Policy Office (Belspo) for financial supporting through the Inter-University Attraction Poles (IAP) “AquaStress” project. TK thanks Vietnamese Government for financial supporting through Vietnamese Students Postgraduate in Foreign Countries Programme (VIED). FR-L thanks Fondecyt regular grant (project number 1211841; ANID; Government of Chile).

## Conflict of Interest

FER-L is Senior Research Associate for Ictio Biotechnologies S.A.

The remaining authors declare that the research was conducted in the absence of any commercial or financial relationships that could be construed as a potential conflict of interest.
